# Comparative evaluation of force decay pattern in orthodontic active tiebacks exposed to five different mouth rinses: An in vitro Study

**DOI:** 10.34172/joddd.2020.048

**Published:** 2020-12-09

**Authors:** Amir Hossein Mirhashemi, Atefe Saffar Shahroudi, Keyvan Shahpoorzadeh, Niloofar Habibi Khameneh

**Affiliations:** ^1^Department of Orthodontics, School of Dentistry, Tehran University of Medical Sciences, Tehran, Iran; ^2^Dental Research Center, Dentistry Research Institute, and Department of Orthodontics, Dental School, Tehran University of Medical Sciences, Tehran, Iran; ^3^School of Dentistry, Tehran University of Medical Sciences, Tehran, Iran

**Keywords:** Active tiebacks, Force degradation, Mouth rinse, Orthodontics

## Abstract

**Background.** This study compared the force decay pattern of two different orthodontic active tiebacks (ATBs) exposed to five different commercially available mouth rinses.

**Methods.** In this in vitro study, 90 transparent ATBs and 90 gray ATBs were divided into six groups; one was the control group, and the others were exposed to one of these mouth rinses twice a day for 60 seconds: Listerine, chlorhexidine, Orthokin, Persica, and fluoride. The initial force of each ATB was 250 g at a 24-mm extension. The force of ATBs was measured on days 1, 7, 14, and 28 using a digital gauge.

**Results.** The highest percentage of force loss was observed between days 14 and 28 (P<0.05). At the end of the study, the Persica group exhibited the highest force degradation in both ATB types. In the transparent ATBs, it was followed by Orthokin, Listerine, fluoride, chlorhexidine, and control groups, respectively. In the gray ATBs, Orthokin, chlorhexidine, control, Listerine, and fluoride groups exhibited the highest force decay in descending order. In some groups, the differences between transparent and gray ATBs were significant. In the control group, the force of transparent ATB was significantly higher than gray ones on days 7 and 14 but not significantly after four weeks.

**Conclusion.** ATBs’ force degradation could be exacerbated by the use of some mouth rinses. There were some differences between force relaxation patterns of transparent and gray ATBs. The data could be beneficial in choosing appropriate O-rings for making ATBs.

## Introduction

Different space closure systems have been proposed in orthodontic treatment, including elastomeric products, such as elastomeric chains and modules, and nickel-titanium coil springs.^1^ In order for a space closure system to be ideal, it should have mechanical properties that provide a continuous light force with minimal decay over time.^2^ Although elastomeric products are more frequently used because of the simplicity of handling, low chair time, and low cost, they have the disadvantages of undergoing force degradation over time, resulting in a decrease in tooth movement rate and elongation of the treatment process.^3^ The greatest degree of force loss occurs in the first three hours with a relatively steady force loss rate in the following days, and this rate has been shown to be faster in water than dry conditions and could be affected by the chemicals in the solutions.^4,5^

Several studies have addressed the effect of different environmental changes and various solutions on the elastomeric chains’ force decay pattern. These solutions were disinfectants or mouth rinses.^6-11^ The application of mouth rinses is of great importance for orthodontic patients because of the higher risk of plaque accumulation around fixed orthodontic appliances and subsequently greater risk of accumulation of cariogenic bacteria in their oral environment.^12,13^ The effect of commonly used mouthwashes, such as chlorhexidine,^14^ sodium fluoride,^11^ Listerine, Persica,^9,15^ and Orthokin^16^ on the force of elastic chains and their potential, influential factors, such as the pH of the mouth rinses,^17^ bleaching agents,^10^ and alcohol content^7^ have been investigated. For example, it was shown that chlorhexidine and sodium fluoride caused a higher force loss in elastomeric chains than the control group, while Persica reduced the amount of force decay.^9^ Apart from the environmental factors, some intrinsic characteristics of elastomeric products, such as the chains’ color, can also affect their relaxation pattern.^2^ Therefore, it is possible that elastomeric products with different colors from the same manufacturer respond differently to chemical solutions, and thus it is prudent to compare the force decay patterns of different colors of elastomers exposed to mouth rinses to achieve better clinical results.

Elastomeric modules are a kind of orthodontic elastomeric product that are available in various shapes and colors. Their force decay pattern and the effect of a different chemical on that has also been investigated.^18^ Apart from their role as the ligature of the archwire in braces, they have been used to make active tiebacks (ATBs). ATBs were popularized by McLaughlin and Bennett^19^ in the late 1980s as a mechanism for space closure with pre-adjusted straight-wire appliances. They refer to the use of stainless steel ligatures threaded through an elastic module that goes directly from the terminal molar to the canine bracket. A study showed that they exerted more appropriate initial force and slower force decay than elastic chains.^20^

Although many researchers have investigated the effect of mouth rinses on force degradation of orthodontic elastomeric materials, most studies have evaluated elastomeric chains, and few have considered ATBs.^21^ Accordingly, this study aimed to compare the force decay pattern of two different orthodontic ATBs made out of elastomeric modules with different colors, which were exposed to five different commercially available mouth rinses over time, including Listerine, Chlorhexidine, Orthokin, Persica, and fluoride.

## Methods

**Figure 1:**
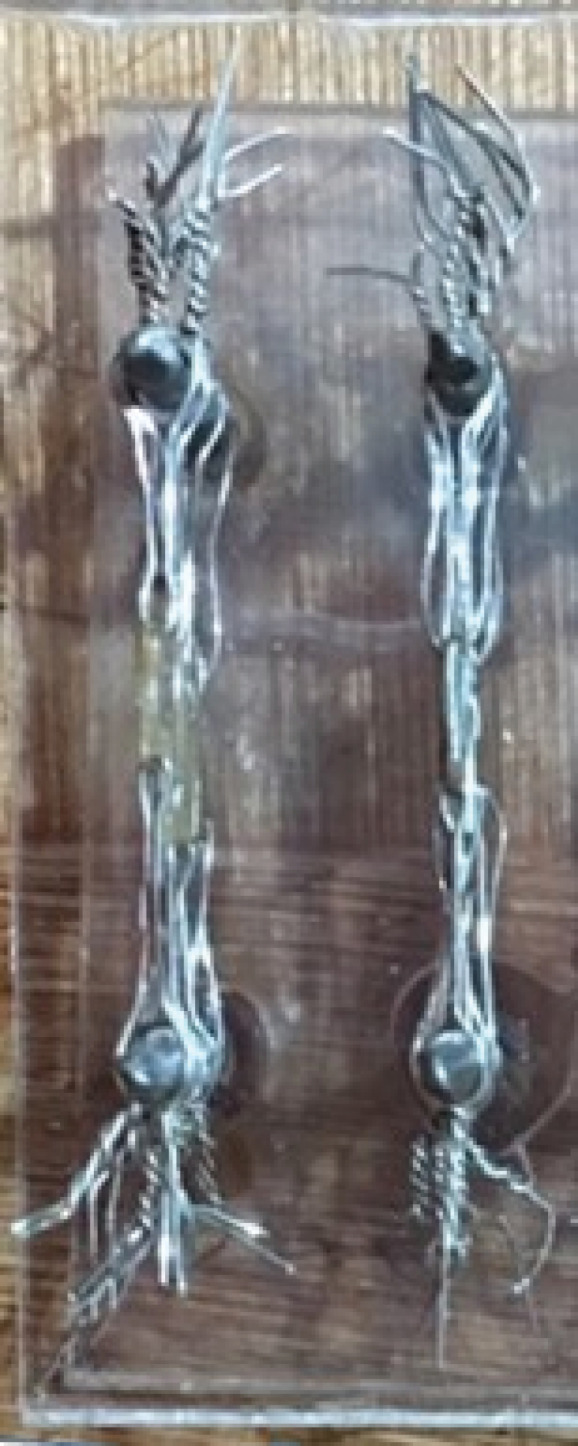
The two types of active tiebacks were stretched so that they exerted 250 g force initially at the 24 mm extension.

**Figure 2:**
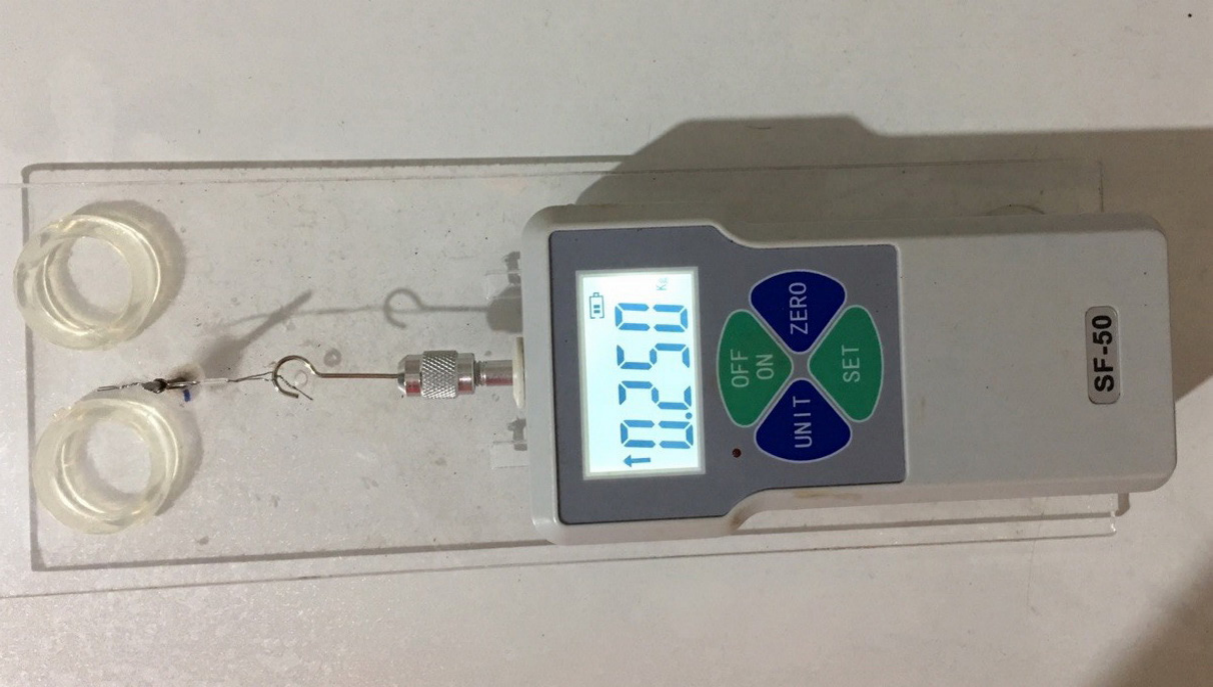
The force was measured using a digital force gauge.

In this in vitro study, the effect of 0.2% fluoride, 0.2% chlorhexidine, Persica, Orthokin, and Listerine mouthwashes on the force exerted by two types of ATBs was evaluated. Therefore, twelve groups were tested with a total sample size of 180 specimens, including two control groups. The ATBs were made of transparent and gray elastomeric modules (American Orthodontics, USA). A custom-made jig was designed with a series of pins that held the stretched ATBs at a fixed length of 24 mm. This distance was considered as an average distance between the canine and first molar teeth.^21^ The ATBs were mounted on these jigs, and the stainless steel ligatures were threaded through an elastic module and twisted so that they exerted a 250-g force at the 24-mm extension (Figure 1). The force was measured with a digital force gauge (model: SF-50, Germany) (Figure 2). Six jigs were prepared, each containing two rows of pins, one row for gray ATB specimens and one row for transparent ones. Fifteen ATB specimens were mounted on each row.

To simulate oral conditions, all the six jigs were immersed in artificial saliva and stored in an incubator at body temperature (37±1°C). One jig was considered as a control group, and the other five groups were exposed to one of the studied mouth rinses: 1) 0.2% chlorhexidine (Behsa Pharmaceutical Company, Arak, Iran) containing 0.2 g of chlorhexidine gluconate in every 100 mL of solution; 2) 0.2% sodium fluoride (Behsa Pharmaceutical Company, Arak, Iran) containing 0.2 g of sodium fluoride in every 100 mL of solution; 3) Persica mouthwash (Poursina Pharmaceutical Company, Tehran, Iran) containing miswak plant, mint, and yarrow active ingredients. The most important organic and mineral ingredients of the drop are tannins, flavonoids, flavoring agents, calcium, fluoride, and chloride; 4) Total Care Zero Listerine mouthwash (Johnson and Johnson, Italy); 5) Orthokin alcohol-free mouthwash (Kin, Spain).

The specimens were soaked in the relevant mouthwashes twice a day for 60 seconds each time with a 12-hour interval. Following each immersion, the samples were washed for 10 seconds with distilled water before being transferred to the artificial saliva and incubator again. The force exerted by each group was measured using a digital force gauge (model: SF-50, Germany) in Newton and gram units with up to 1 g accuracy, at 0, 1-day, 7-day, 14-day, and 28-day intervals.

Three-way ANOVA was used to compare the effect of time and mouth rinse type on the force exerted by ATBs. However, since none of the triple and double interactions were significant, each group was analyzed with one-way ANOVA and post hoc Tukey tests.

## Results

In this study, the mean initial force for all the specimens at a 24-mm extension was 249.8±0.6 g. The force exerted by transparent and gray ATBs in different mouth rinse groups at different time intervals is presented in Tables 1 and 2, respectively. The percentage of remaining force relative to the initial force is also given in parentheses.

**Table 1:** Mean forces in gram and the remaining force percentage (numbers in parentheses) in transparent O-rings at different time intervals in different mouth rinse groups during the study

**MR**	**Day 0**	**Day 1**	**Day 7**	**Day 14**	**Day 28**
Control	249.8 (100)	195.2 (78.1)	188.4 (75.4)	186.6 (74.6)	145.6^a^ (58.2)
Persica	249.4 (100)	189.6 (76.0)	177.6^ab^ (71.2)	175.6^ab^ (70.4)	122.7^ab^ (49.1)
Fluoride	250.8 (100)	188.4 (75.4)	187.8 (74.8)	185.6 (74.0)	132.8^a^ (52.9)
Chlorhexidine	249.8 (100)	188.3 (75.3)	187.2 (74.9)	185.0 (74.0)	138.8^a^ (55.5)
Orthokin	250.6 (100)	192.6 (76.8)	187.3 (74.7)	186.0 (74.2)	125.0^ab^ (49.8)
Listerine	249.6 (100)	185.6 (74.5)	184.0 (73.8)	181.9 (73.0)	126.0^ab^ (50.5)

MR: mouth rinse ^a^ Significantly different from the first follow-up in the same group. ^b^ Significantly different from the control group at the same follow-up time.

**Table 2:** Mean forces in gram and the remaining force percentage (numbers in parentheses) in gray O-rings at different time intervals in different mouth rinse groups during the study

**MR**	**Day 0**	**Day 1**	**Day 7**	**Day 14**	**Day 28**
Control	250.4 (100)	178.0 (71.0)	174.7 (69.7)	170.9 (68.2)	128.7^a^ (51.3)
Persica	249.6 (100)	179.7 (71.9)	173.0 (69.3)	170.6 (68.3)	101.1^ab^ (40.5)
Fluoride	249.1 (100)	174.5 (70.0)	170.2 (68.3)	172.6 (69.2)	135.5^a^ (54.3)
Chlorhexidine	249.7 (100)	172.6 (69.1)	161.5 (64.6)	161.2 (64.5)	126.7^a^ (50.7)
Orthokin	249.4 (100)	180.2 (72.2)	181.0 (72.5)	179 (71.7)	106.2^ab^ (42.5)
Listerine	250.0 (100)	176.7 (70.6)	171.2 (68.4)	169.1 (67.6)	131.2^a^ (52.4)

MR: mouth rinse. ^a^ Significantly different from the first follow-up in the same group. ^b^ Significantly different from the control group at the same follow-up time.

### Effect of time on ATBs’ force

There was a steady decrease in the force exerted by all the specimens over time, indicating that the measured force was less than the previous follow-up in all the follow-ups. However, in the control group of both ATB groups, only after 28 days, the mean force was significantly lower than the initial force and also lower than the 14th-day and 7th-day follow-ups (*P* < 0.001). The differences between other follow-up results were not significant.

In the Persica group of transparent ATBs, the force degradation was significant even in the second follow-up after seven days (*P* < 0.001).However, the results of the 14th-day follow-up were not statistically different from the seventh day (*P* = 0.904); however, the final follow-up results were significantly lower than the previous ones (*P*< 0.001).

In the Persica group of the gray ATBs and both transparent and gray ATBs of Listerine, Orthokin, chlorhexidine, and fluoride groups, the exerted force was not significant between the seventh-day and 14th-day follow-ups. However, the degradation rate increased from the third to the last follow-up so that the measured force on the 28th day was significantly lower than all the previous measurements (*P* < 0.001).

### Effect of mouth rinse on ATBs’ force

The statistical analysis showed no significant differences between mouth rinse groups in both transparent (*P* = 0.933) and gray (*P* = 0.778) ATBs on the first day.

On the seventh day, the difference between some mouthwash groups in the transparent ATBs was statistically significant, with the Persica group exhibiting significantly lower force than all the other groups (*P* < 0.001) except Listerine (*P* = 0.106). The Persica group exhibited the lowest force (189.6 g).

In gray ATBs, the difference between the chlorhexidine and Orthokin groups was significant (*P* = 0.02). The chlorhexidine group exhibited the lowest force, while the Orthokin group had the highest force of all the groups, including the control group (Table 2). A similar pattern was observed on the 14th-day follow-up in both the ATBs group.

At the last follow-up on the 28th day in transparent ATBs, the control group exerted the highest force with a significant difference from Persica, Orthokin, and Listerine (*P* < 0.05). The chlorhexidine group also exerted significantly higher force than the Persica group (*P* = 0.023), followed by fluoride, Listerine, and Orthokin groups. Among gray ATBs, Persica exhibited the lowest force of all, and its difference was only not significant with Orthokin (*P* = 0.98). The fluoride group exhibited the highest force, followed by Listerine, control, chlorhexidine, and Orthokin groups.

### Comparison of the two colors of ATBs

A comparison of ATBs’ average forces (Tables 1 and 2) showed that the gray ATBs exerted lower force than transparent ATBs except on the 28th day in the Listerine and fluoride groups in all the follow-ups. However, this difference was only significant on the first day in the chlorhexidine group and on the 7th day in the control, fluoride, chlorhexidine, and Listerine groups and on the 28th day in the Persica and Orthokin groups(*P* < 0.05) (Table 3).

**Table 3:** *P* values of the comparison between the mean forces of transparent and gray O-rings at different time intervals^a^

	**Control**	**Persica**	**Fluoride**	**Chlorhexidine**	**Orthokin**	**Listerine**
Day 1	0.67	0.115	0.45	0.014	0.155	0.383
Day 7	<0.001	0.532	<0.001	<0.001	0.141	<0.001
Day 14	0.002	0.195	<0.001	<0.001	0.068	<0.001
Day 28	0.135	<0.001	0.979	0.175	0.001	0.917

In all the significant comparisons, the mean force of transparent O-rings was higher than the gray ones. ^a^ The level of significance was set at *P* < 0.05.

## Discussion

Orthodontic patients are more prone to dental caries and gingival problems due to a higher rate of plaque accumulation. Accordingly, they are mostly prescribed different antibacterial agents in the form of mouth rinses, toothpastes, varnishes, and so on.^22,23^ On the other hand, in most orthodontic treatments, elastomeric products are applied for force exertion. Concerned about the potential adverse effects of these chemical agents on the force of elastomeric products and subsequently on the efficacy of orthodontic tooth movement, this study was conducted to evaluate the effect of some common mouth rinses, including 0.2% fluoride, 0.2% chlorhexidine, Orthokin, Listerine, and Persica on the force of ATBs.

This study showed that ATBs underwent force degradation over time, which could be influenced by the mouth rinse type and orthodontic elastomeric modules’ color. The follow-ups of the study lasted up to 28 days, which accounts for a common time interval between two orthodontic appointments for changing ATBs;^10,14,24^ however, some studies have considered 3-week follow-ups.^25,26^ In the current study, the initial force was set at about 250 gr. The optimal force in canine retraction was considered at 100‒300 g.^27^ Although in some previous studies on elastomeric chains, 200 g of force was applied,^5,8,15^ we preferred to apply higher force since the ATBs are usually used in the McLaughlin and Bennett pre-adjusted straight-wire system for en-masse retraction of the six front teeth simultaneously rather than canine retraction alone.^19^ The custom-made jigs fabricated to keep the ATBs stretched at a length of 24 mm, suggested by previous studies as an average distance between canine and first molar teeth.^21^

Although many studies have reported the force degradation of elastomeric chains over time, very few studies have addressed ATBs’ force loss pattern.^20,21,28^ Mohammadi and Mahmoodi ^28^ compared the force decay pattern of elastomeric ligatures and elastomeric separators of three different manufacturers (Dentaurum, RMO, 3M Unitek) in an ATB state in a simulated oral environment. Elastomeric ligatures and elastomeric separators were stretched to 100% and 150% of their original inner diameter and the force level was measured at different time intervals. They concluded that the force decay pattern was relatively identical in all the products with a force loss of 62%‒81% in 4 weeks. In another study, the amount of force decay between the elastomeric chain and tie-back method over a 48-hour period was compared. Elastomeric chains were stretched 100% of their initial length, and elastic modules in the tieback method were stretched twice their original diameter. It was concluded that elastic modules had a lower initial force than the elastomeric chains (with a mean force of 577.50 g vs. 650.00 gr, but elastomeric chains showed a significantly higher force decay over time (446.50 g vs. 209 g).^20^

Regarding the effect of mouth rinses on the force decay pattern of elastomeric products, several studies have evaluated power chains, but few have assessed ATBs. Menon et al^8^ studied the effect of Listerine, Clohex Plus with a chlorhexidine base, and Colgate Phos-Flur with a fluoride base on elastomeric chains. They found that Listerine mouthwash exhibited the most force degradation of all (71.61%), followed by Colgate Phos-Flur mouthwash (65.22%) and Clohex Plus with a chlorhexidine base (64.91%).^8^ Similarly, in the current study, Listerine mouthwash was associated with higher force degradation of ATBs than fluoride and chlorhexidine mouthwashes. In both studies, the time duration was four weeks, while the amount of force decay was different. Relatively similar results about Listerine were reported by Pithon et al,^10^ who investigated the effect of mouthwashes with and without bleaching agents on elastomeric chains and found that Listerine mouthwash (with or without bleaching agents) led to statistically higher force degradation than the control group.

Regarding other mouthwashes, Omidkhoda et al^9^ studied the effect of Persica, chlorhexidine, and fluoride mouthwashes on the elastomeric chains’ force degradation. The study was performed in 28 days, similar to the present study, and it was reported that fluoride mouthwash caused 46.05% and 56.81% force degradation in the short-connector and closed-connector elastomeric chains, respectively. Chlorhexidine mouthwash caused 49.64% and 59.85% force degradation in the mentioned elastomeric chains, and Persica caused 40.31% and 43.19% force degradation.^9^ A comparison of the results above with the current study results showed that the fluoride group had an almost similar force degradation pattern in both studies. In contrast, the chlorhexidine group exhibited less and the Persica group had more force degradation in ATBs than elastomeric chains. However, the statistical significance of this comparison has not been analyzed. The initial force was 200 g in their study, which is different from the present study with 250 g.

Oshagh et al^21^ studied the effect of chlorhexidine and fluoride mouthwashes on elastomeric chains and ATBs and Ni-Ti coil springs. They found that the fluoride mouthwash caused more force degradation than chlorhexidine, and chlorhexidine caused more force degradation than the control group in elastomeric chains. The highest force degradation was recorded in the chlorhexidine group in ATBs, followed by the fluoride and control groups. Similarly, in the present study, the chlorhexidine group exhibited the highest force degradation in gray ATBs, followed by the fluoride and control groups. However, in transparent ATBs, the fluoride group exhibited higher force degradation than the chlorhexidine group. In both studies, the mouthwash groups exhibited more force degradation than the control group. The study’s duration was three weeks in Oshag and colleagues’ study, which was shorter than the current study, and the samples were exposed to the relevant mouthwashes once a day compared to this study, which was twice a day. The storage solution was distilled water rather than artificial saliva.

The effect of Orthokin, Sensikin, and Persica on force degradation of elastomeric chains and coil springs was compared in a study by Javanmardi and Salehi.^16^ They reported a 45.42% force degradation after three weeks of incremental exposure to Orthokin, which is comparable to 50% and 57.52% force degradation in transparent and gray ATBs of the present study, respectively.^16^ Persica had more force degradation than Orthokin in both studies, and it could be concluded that ATBs and elastomeric chains would show relatively similar behavior when exposed to the same chemicals.

A comparison of the force degradation pattern of the currents study’s groups showed that Persica accelerated the force loss of ATBs with a higher rate than the other mouth rinses in both types of ATBs. This should be noticed by clinicians who prescribe this mouthwash, and shortening the intervals of ATBs’ replacement to <4 weeks is prudent. After Persica, came Orthokin and Listerine in gray ATBs, respectively, with significantly more force degradation than the control group. However, the force degradation of the latter two was not noticeable up to the 14th day, and most force degradation was observed between days 14 and 28. Considering the typical orthodontic follow-ups (3 to 4 weeks), it does not seem that applying these mouth rinses necessitates increasing the number of treatment follow-ups.

It is also worth mentioning that the force exerted by the samples of Listerine and fluoride groups was higher than the control groups on the 28th day in gray ATBs. This can be related to the different pH of these mouthwashes. Thus, it can be suggested that when these mouth rinses should be prescribed for patients for four weeks, it would be better to choose gray ATBs instead of transparent ones. However, more precise studies are recommended on this issue.

A comparison of two ATB types showed that gray ATBs exhibited more force degradation than transparent ones except in the presence of Listerine and fluoride mouthwashes. It was also revealed that gray ATBs had more force degradation than transparent ones in the chlorhexidine mouthwash groups after one day. From the first day to the 14th day, the same pattern was observed in the control, Listerine, and fluoride groups. From the 14th day to the 28th day, in the Orthokin and Persica mouth rinse groups, there was a significant difference between the two ATB types.

If the follow-up intervals are set at four weeks, and Persica or Orthokin are prescribed, or if the follow-up intervals are set at three weeks, and one of the Listerine, fluoride, and chlorhexidine mouth rinses is prescribed, transparent ATB is preferred to the gray one.

There is no preference between transparent and gray ATBs after four weeks when no mouth rinse is used. However, when the orthodontist prefers to change the ATBs after three weeks, transparent ATBs could be a better choice than the gray ones.

This study was a new step in recognizing ATBs’ characteristics. Therefore, more clinical research on ATB’s behavior and its associated factors is strongly suggested.

## Conclusion

ATBs underwent force degradation over time, which was exacerbated by the use of some mouth rinses. The Persica mouth rinse caused the highest force degradation in ATBs.

Chlorhexidine mouthwash caused the least force degradation among all the groups except the control group in transparent ATBs, while in gray ATBs, Listerine and fluoride mouth rinses caused the least force degradation between all the groups, even in the control group.

If Orthokin or Persica mouthwash is prescribed for the patient, and the visits are once in four weeks, it is suggested that transparent ATBs be used.

In all the follow-ups, gray ATBs exerted lower force than transparent ATBs except on the 28th day in the Listerine and fluoride groups. However, their differences were not significant in all the comparisons. In the control group, the difference was significant on the seventh and 14th day and not on the first day or after four weeks.

## Authors’ Contributions

AHM developed the idea of research and coordinated the project and was the advising professor. ASS was the co-advisor and carried out the scientific writing of the article. KS and NHK prepared the samples and conducted the experiments and measurements.

## Funding

This research was a part of an MS thesis with the reference number of 6415, conducted in and funded by the Dental School of Tehran University of Medical Sciences.

## Ethics Approval

Not applicable. This study did not involve human participants or animals.

## Competing Interests

The authors declare no competing interest.
